# Two muscular variations in the elbow associated with the anterior interosseous nerve

**DOI:** 10.1007/s00276-021-02706-9

**Published:** 2021-02-15

**Authors:** Łukasz Olewnik, Bartłomiej Szewczyk, Nicol Zielinska, Dariusz Grzelecki, Michał Polguj

**Affiliations:** 1 Department of Anatomical Dissection and Donation, Medical University of Lodz, Łódź, Poland; 2 Department of Normal and Clinical Anatomy, Medical University of Lodz, Łódź, Poland; 3 Department of Orthopedics and Rheumooorthopedics, Centre of Postgraduare Medical Education, Otwock, Poland

**Keywords:** Flexor pollicis longius, Pronator teres, Anterior interosseous nerve, Median nerve, Case report

## Abstract

The coexistence of different muscular-neurovascular variations is of significant clinical importance. A male cadaver, 76 years old at death, was subjected to routine anatomical dissection; the procedure was performed for research and teaching purposes at the Department of Anatomical Dissection and Donation, Medical University of Lodz. The right forearm and hand were dissected using standard techniques according to a strictly specified protocol. The presence accessory head of the flexor pollicis longus may potentially compress the anterior interosseous nerve. The present case report describes a rare variant of the ulnar head of the pronator teres, characterized by two independent bands (i.e., two proximal attachments). The main band originates from the coronoid process and the second originates from the tendon of the biceps brachii. This type of attachment could potentially affect the compression of the ulnar artery running between the two bands. Additionally, the accessory head of the flexor pollicis longus was observed, which started on the medial epicondyle; its coexistence with a high division median nerve creates a potential pressure site on the anterior interesosseous nerve.

## Introduction

Morphological variations can affect routine clinical procedures or important and complex surgeries. For example, the occurrence of a coracobrachialis longus muscle may influence potential compression on the median nerve, ulnar nerve or musculocutaneous nerve [22, 23]. A similar situation can occur at the site of the median nerve ran between the two pronator teres heads, as well as the site where the median nerve crosses the palmaris longus tendon [20, 21]. The accessory head of the flexor pollicis longus (ahFPL) may lead to entrapment neuropathy of the anterior interosseous nerve (AIN) [1, 2, 6, 9, 13, 15, 19, 25].

Earlier studies on the morphological variability of the pronator teres (PT) focused mainly on the incidence of the ulnar head [7, 11, 14, 18, 21, 28] or its relationship to the median nerve [7, 11, 14, 18, 21, 28]. In contrast, variations in the flexor pollicis longus mainly concerned its morphological variability, and studies focused on the incidence of composite accessory heads and place of origin [1, 2, 6, 9, 13, 15, 19, 25].

This paper describes an extremely rare co-occurrence of a rare variation of the PT ulnar head (uhPT) with one of the accessory head of the flexor pollicis longus (ahFPL). The dual proximal attachment of the uhPT creates a potential pressure point on the ulnar artery. It should be noted that the prevalence of vascular compression in the elbow area is extremely rare: only 1%. Our findings highlight the importance of muscle variants in the forearm region, and are of interest to radiologists, anatomists, physiotherapists and surgeons specializing in the forearm and hand region.

## Case report

A male cadaver, 76 years old at death, was subjected to routine anatomical dissection for research and teaching purposes at the Department of Anatomical Dissection and Donation, Medical University of Lodz. The right forearm and hand were dissected using standard techniques according to a strictly specified protocol [20, 21, 30, 31]. During the dissection of the left forearm, two morphological variations were found.

**Fig. 1.**
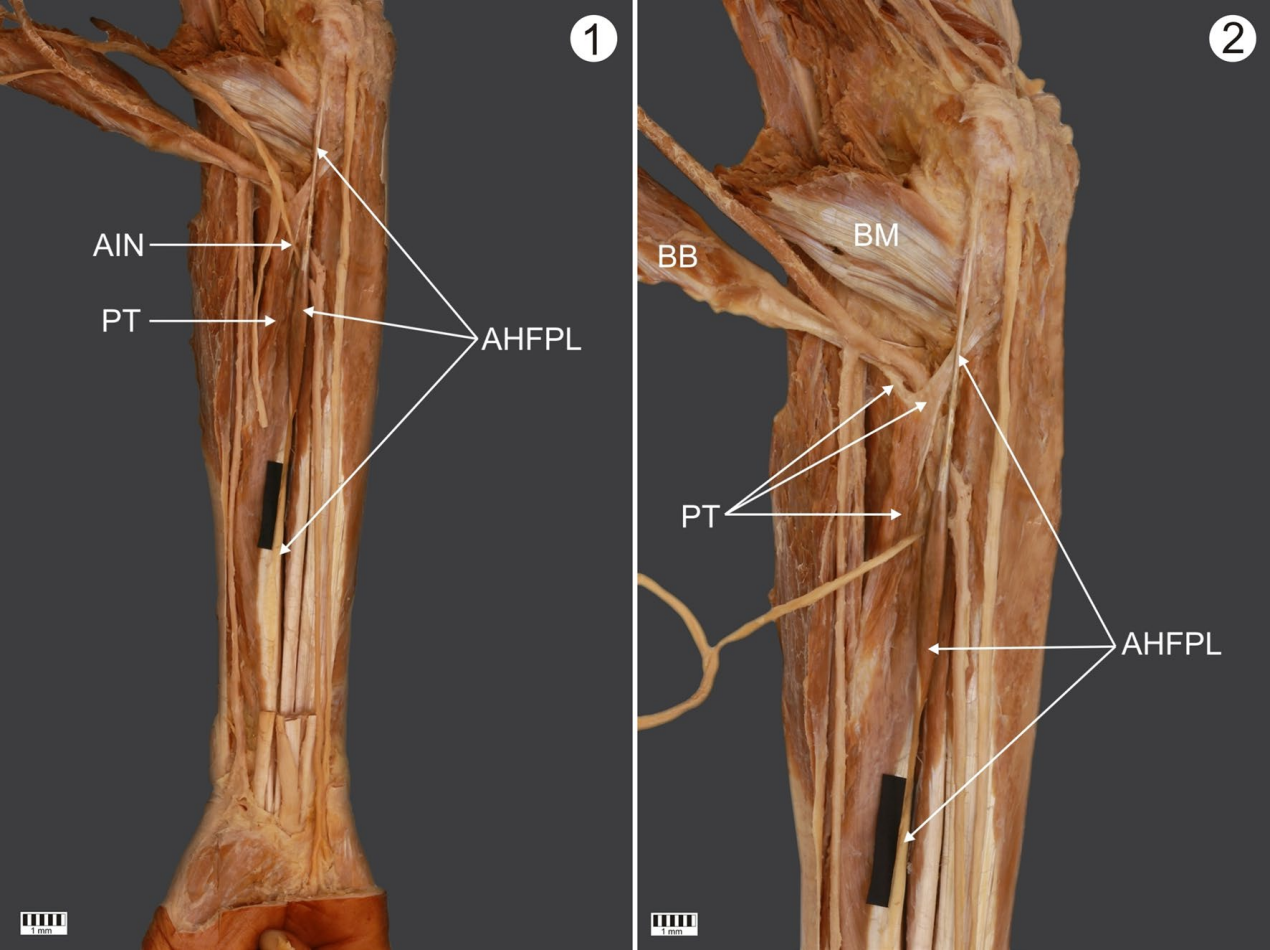
**1** Complete view of the forearm. Right forearm. Anterior view. Visible accessory head of the flexor pollicis longus. *AHFPL* accessory head of the flexor pollicis longus. *PT* pronator teres muscle *AIN* anterior interosseous nerve. **2** Proximal part of the forearm. Anterior view of the right forearm. *BM* brachialis muscle *BB* biceps brachii *AHFPL* accessory head of the flexor pollicis longus *PT* pronator teres muscle

1. Accessory head of the flexor pollicis longus muscle (ahFPL):
  The ahFPL originated from the medial epicondyle of the humerus, under the flexor digitorum superficialis. The origin began with a thin, 78.04 mm-long tendon. The origin of the accessory head was fused with most of the brachialis muscle, as well as the ulnar head of the pronator teres muscle. The proximal tendon passed into a 54.80 mm-long muscle belly; the belly then passed into a 54.14 mm-long distal tendon and inserted on the radial border of the FPL. It was fusiform-shaped—Fig. 1.
2. A rare case of the ulnar head of the pronator teres (uhPT) and its relationship to the ulnar artery

The PT consisted of two independent heads (the humerus and ulna) that did not connect to one another in the distal part. The humeral head was cut to highlight the variability regarding the ulnar head of the PT. The ulnar head originates by two independent bands (two proximal attachments). The main band originated from the coronoid process and the accessory bands from the AHFPL, while the second originated from the tendon of the biceps brachii—Fig. 1. Morphometric measurements of the bands are given in Table 1. Following this, both bands joined together and became the muscle belly; the distal tendon then inserted at the middle of the lateral surface of the radius. A “fibrous arch” can be seen at the junction of the two bands, through which the ulnar artery runs. At the transition point, it has a diameter of 5.09 mm—Fig. 1.

**Table 1:** Morphometric measurements of the proximal attachments of the ulnar head of the PT

	Origin	
	First band	Second band
	Coronoid process	Tendon of the biceps brachii
Length		
Tendinous part	29.13 mm	8.42 mm
Width		
Origin	6.17 mm	2.59 mm
Myotendinous junction	8.19 mm	4.54 mm
Thickness		
Origin	3.37 mm	1.45 mm
Myotendinous junction	4.01 mm	1.67 mm

The measurements were performed using two methods:

- Direct measurements by electronic caliper (Mitutoyo Corporation, Kawasaki-shi, Kanagawa, Japan). Each measurement was carried out twice with an accuracy of up to 0.1 mm.
- Analysis of digital photographic images processed through MultiScanBase 18.03 (Computer Scanning System II, Warsaw, Poland).

## Discussion

Our case illustrates an extremely rare co-occurrence of a rare variety of PT ulnar head and an accessory head of the flexor pollicis longus. The uhPT originates by two independent bands (two proximal attachments): the main one originates from the coronoid process and the other from the biceps brachii tendon. This arrangement is associated with a potential risk of compression on the ulnar artery. The ahFPL originates from the medial epicondyle of the humerus, under the flexor digitorum superficialis.

Morphological variations in PT occur quite frequently and most often concern the presence or absence of uhPT or the relationship of the whole muscle to the course of the MN [4, 7, 11, 21, 26, 28]. The frequency of occurrence of the uhPT has been found to vary greatly between studies. Its absence has been noted in 10.8% of cases in one study[11], in 15% in another [26], as well as in 22% [18], in 14% [7] [21], and in one out of 30 cases [4]. Interestingly, none of these studies noticed the lack of a humeral head of the PT [4, 7, 11, 14, 18, 21, 26, 28]. Testut [28] and LeDouble [14] report that the humeral head of the PT may be completely separate. The PT was found to present typical independent heads in the present case. Cases involving the third head of the PT have also been described in the literature [3]. The course of the MN in relation to PT depends on the morphology of the muscle and its adjacent anatomical structures (Table 2) [11, 17, 18, 21].

**Table 2:** Comparison of the course of the median nerve in relation to the pronator teres muscle

Course of median nerve	Mori	Nebot-Cegarra et al.	Jamieson and Anson	Olewnik et al.
Between both heads	95%	75%	83.3%	74%
Beneath both heads	0.25%	–	6%	12%
Beneath HH	–	21.6%	8.7%	14%
Through UH	–	3.4%	–	–
Duplicate HH, through HH	0.25%	1.7%	2%	–
Together with UA	–	–	–	–

*HH* humeral head, *UH* ulnar head, *UA* ulnar artery

The uhPT originates by two independent bands (two proximal attachments). The main band originates from the coronoid process and the second from the biceps brachii tendon; the latter can compress the ulnar artery. Interestingly, entrapment syndrome is very rare in this region. The prevalence of vascular compression in the elbow area is only 1% [5, 8, 27]. Talha et al. [27] describe a case of brachial artery entrapment caused by compression by the supracondylar process, Blakeborough et al. [5] report ulnar artery entrapment by the ligament of Struthers, while Chemla et al. [8] describe two cases of forearm arterial entrapment syndrome in patients having angioaccesses for hemodialysis: one case was due to a fibrosis arcade arising from the flexor digitorum superficialis muscle and the other was due to pronator teres muscle syndrome. Both entrapment syndromes were revealed by angioaccess iterative thromboses [8]. Talha et al. [27], Blakeborough et al. [5] and Chemla et al. [8] report no neurological complaints during the compression.

In the present case reports, the uhPT was characterized by the presence of two proximal attachments with a "fibrous arch" formed at the junction of the two bands; this fibrous arch provides passage for the ulnar artery. At the transition point, it has a diameter of 5.09 mm. This is a potential site of compression on the ulnar artery. But as vascular compression is largely asymptomatic, it is rarely reported.

Despite playing an important role in the potential compression on nerves, arteries or veins, muscular coexistence is very rarely described in the literature [23, 24]. The present report describes the coexistence of a rare uhPT variety with an accessory head of the flexor pollicis longus. The presence of this uhPT type, i.e., originating with a single band from the biceps brachii, may shift the contraction force from the biceps brachii over the elbow joint, thus helping the biceps brachii flex the elbow. The ahFPL may originate from the medial epicondyle of the humerus, the coronoid process of the ulna or the flexor digitorum superficialis muscle [2, 9, 10, 16, 29]. In the present case report, the ahFPL was found to originate from the medial epincondyle, under the flexor digitorum superficialis. The incidence of the ahFPL is quite high and ranges from 25 to 73.6%. [2, 9, 10, 12, 16, 29]. The ahFPL arises during the development of the common flexor mass. This differentiates into superficial and deep layers, the latter differentiating into the FDP, the flexor pollicis longus and the pronator quadratus muscle. Incomplete cleavage of the deep layer results in the formation of the ahFPL. The FPL is primitive or absent in primates, due to it being incorporated into the flexor muscle mass; in such cases the thumb flexor has no functional independence.

Anterior Interosseous nerve (AIN) syndrome is a rare syndrome that comprises less than 1% of all upper extremity nerve palsies, arising due to compression or inflammation of the AIN of the forearm. The ahFPL may lead to entrapment neuropathy of the AIN. This syndrome is manifested clinically as a weakness in the flexion of the interphalangeal joint of the thumb and the distal interphalangeal joints of the index and middle finger. In the present case report, the AIN ran under the ahFPL in the distal part; this is also a potential site for nerve compression.

## Conclusion

The coexistence of the rare ulnar head of pronator with the accessory head of the flexor pollicis longus are extremely rare. The presence of a double proximal attachment of the pronator teres may additionally put pressure on the ulnar artery.

## Data Availability

Please contact authors for data requests (Łukasz Olewnik PhD—email address: lukasz.olewnik@umed.lodz.pl).
